# Supplementary material for: Hollow fiber-combined glucose-responsive gel technology as an in vivo electronics-free insulin delivery system
Source: Commun Biol. 2020 Jun 17;3:313. doi: 10.1038/s42003-020-1026-x (PMC7299969; doi:10.1038/s42003-020-1026-x)
Supplement: Supplementary file 1 — Supplementary Information [file 42003_2020_1026_MOESM1_ESM.pdf]

Supplementary Figures

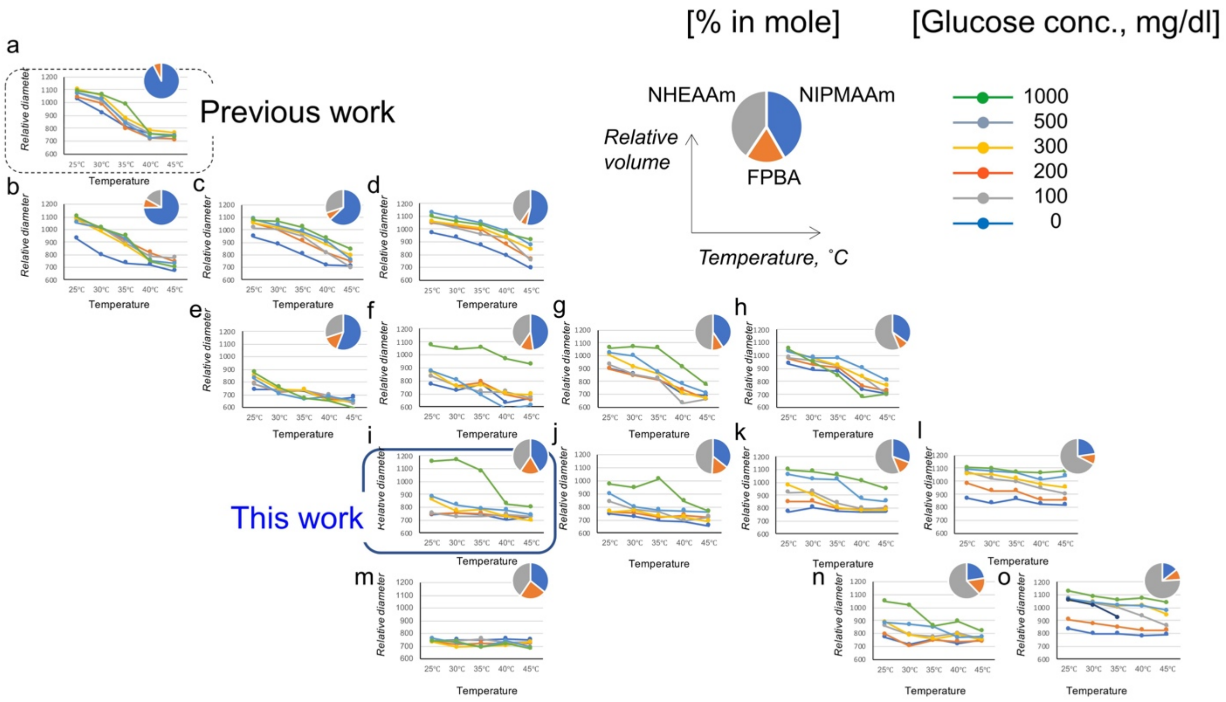

Supplementary Fig. 1. Screening of gel structure for minimized temperature-dependent functionality. Phase diagrams of the gel for different monomer compositions in feed showing their equilibrium volume (hydration) changes as functions of temperature and glucose concentrations investigated at pH 7.4. Pie charts indicate feed compositions of each monomer. Detailed monomer molar fraction can be found in SI Table 1. In each formulation, the gel tends to swell (become more hydrated) with increase of glucose and decrease of temperature. It is also observed that the relative volume of the gel increases (network-loosening) with increased content of NHEAAm, i.e., e→f→g→h or i→j→k→l, and decreases with increased content of FPBA, i.e., or d→f→i→m. With these relationships in mid, our screening criteria was to find a formulation that can (1) minimize the temperature-dependency for the glucose range of 100-500 mg/dL, while also (2) minimizing the network-loosening for lower range of glucose in order to avoid unwanted leakage of insulin. To meet (2), we aimed to achieve comparative dehydration level to that of formulation (a) so that the gel would not compromise on the ability of skin layer formation.

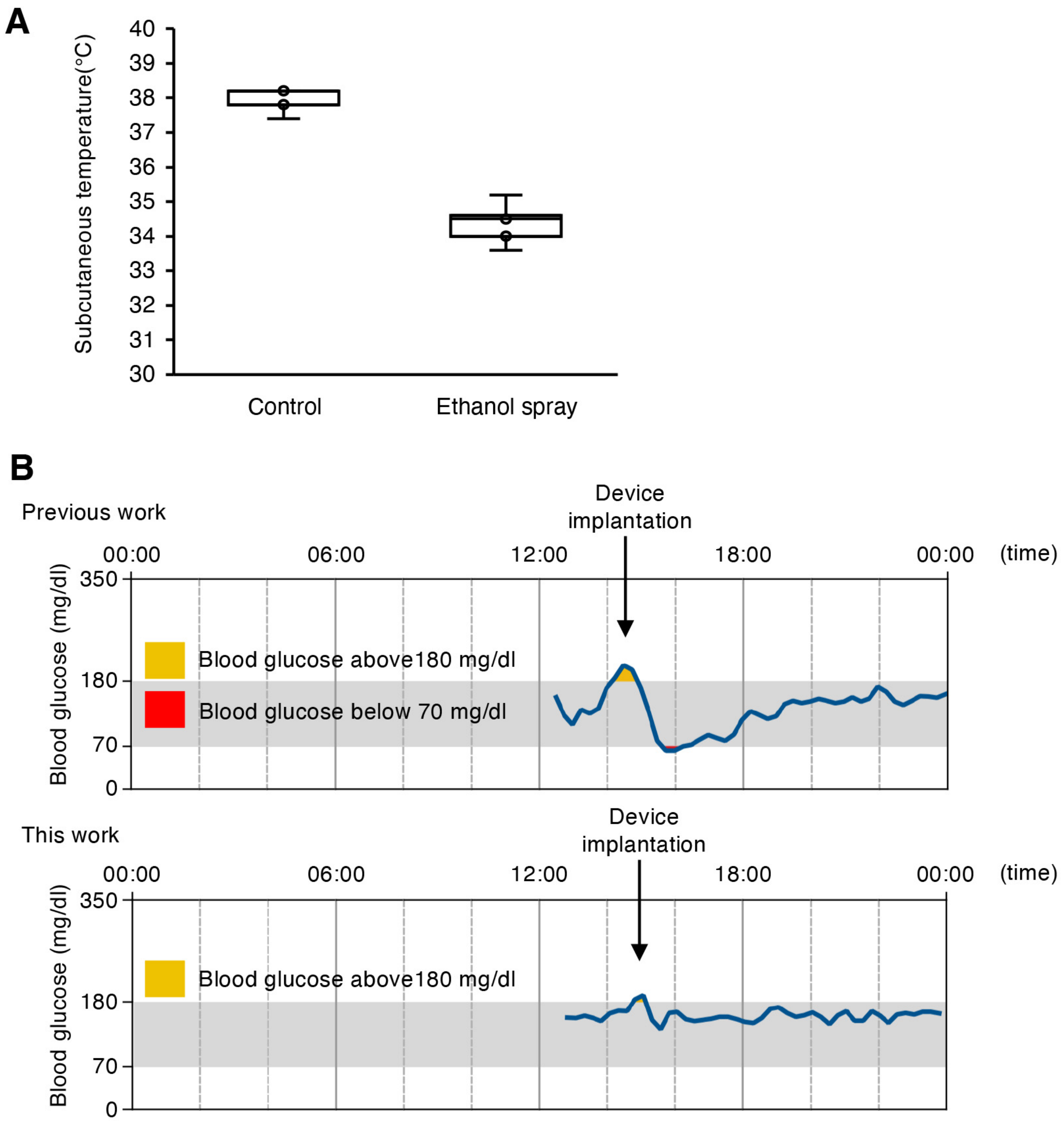

Supplementary Fig. 2. Temperature-independency of the device *in vivo*. Subcutaneous temperature in the control rat (left) and after ethanol spray (right) (A). Each group n = 5. Continuous blood glucose monitoring data intra- and post-implantation of the device; Previous work (upper), This work (lower) (B).

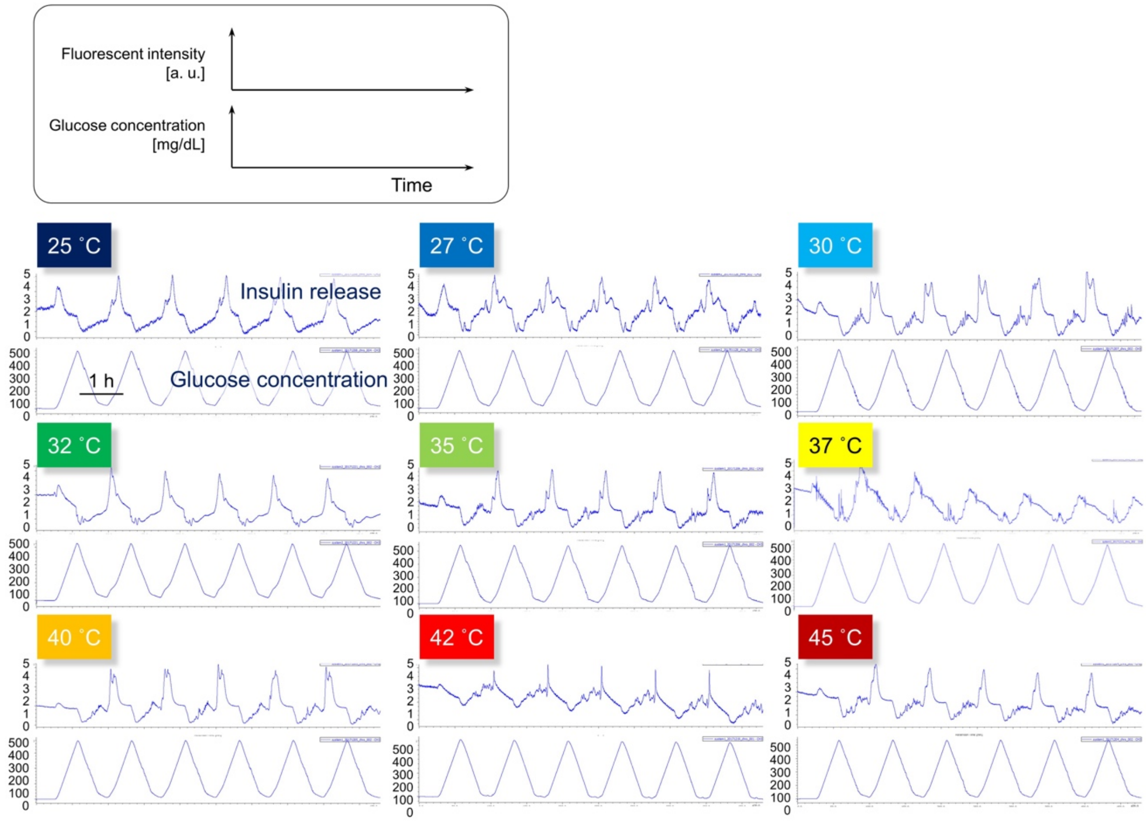

Supplementary Fig. 3. Release experiment under various temperatures. Insulin release experiment using "temperature-insensitive" gel shown in Fig. 2B (blue rectangular).

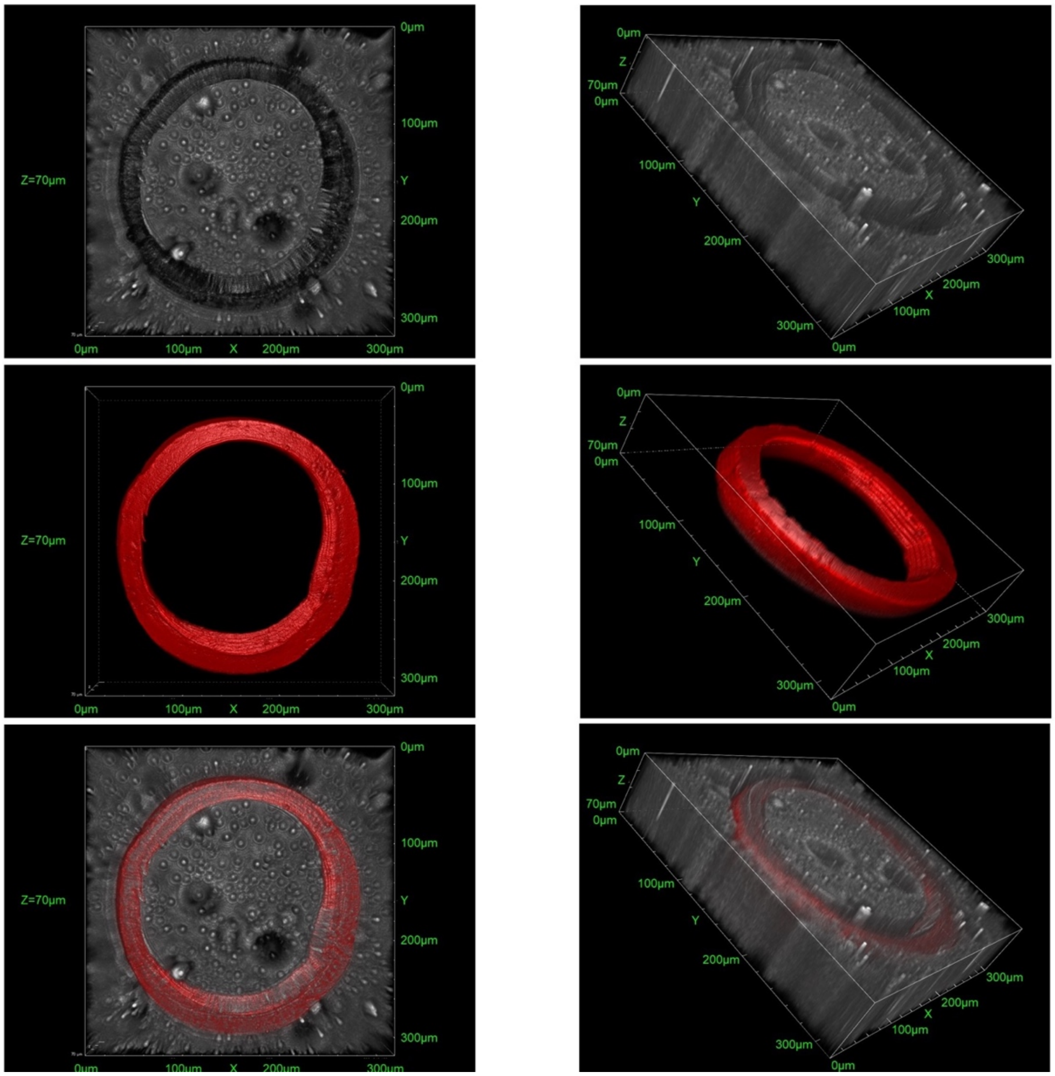

Supplementary Fig. 4. Confocal images of gel-installed fiber section. Top: transmittance image, middle: fluorescence image of Rhodamine-conjugated gel, bottom: superposition of the top and the middle, from different angles (left and right).

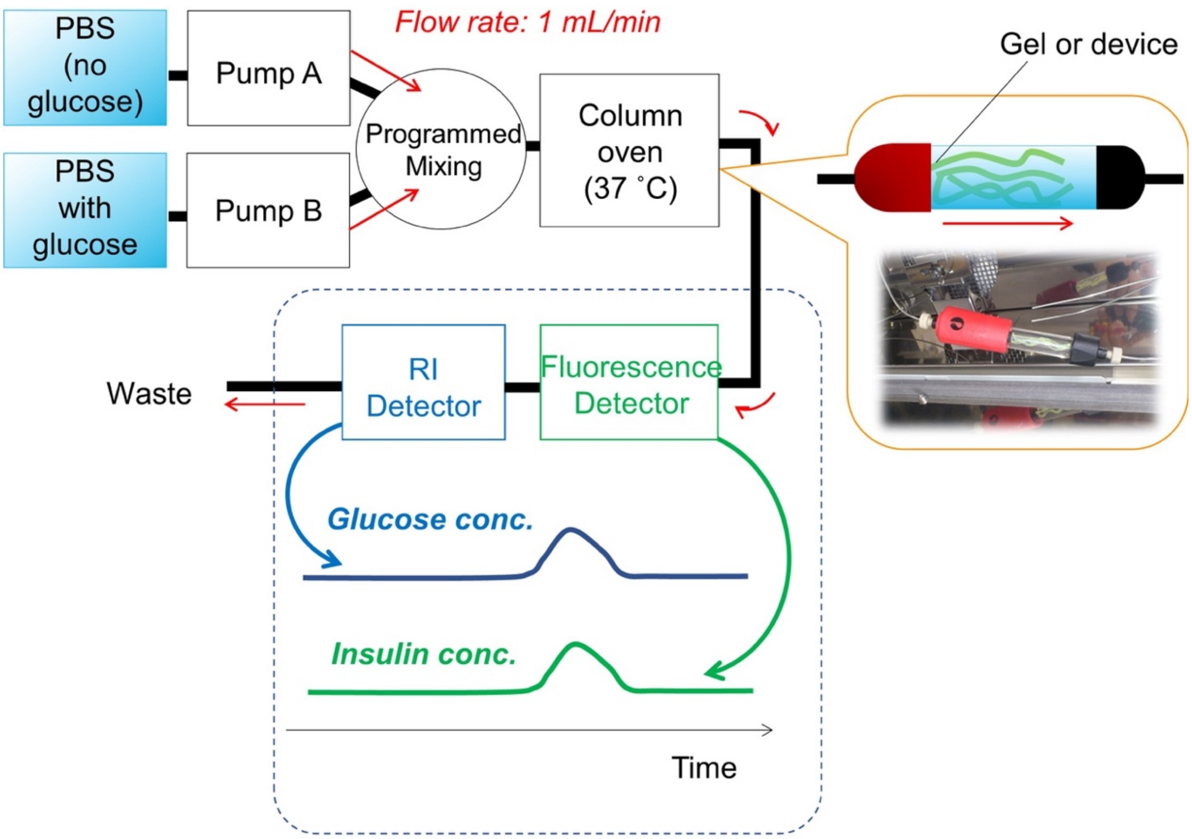

Supplementary Fig. 5. Diagram of HPLC set up used for insulin release experiment. A HPLC system equipped with two pumps and internal detectors; refractive index (RI) for determination of glucose concentration and fluorescence intensities for determination of FITC-labelled insulin concentration.

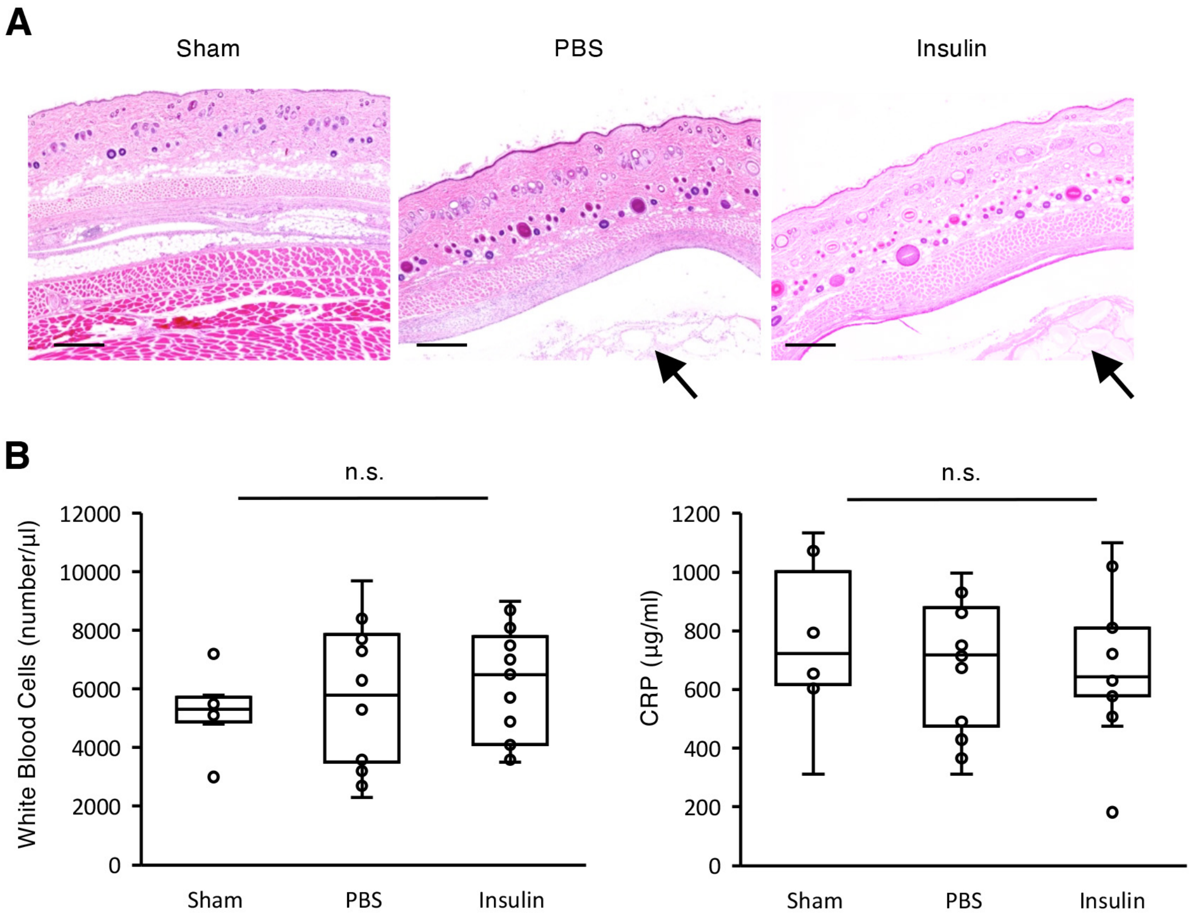

Supplementary Fig. 6 Biosafety of the device *in vivo*. Nine days after implantation in healthy normoglycemic rats, biosafety of the device was evaluated. (**A**) Representative images of the device and the surrounding subcutaneous tissue stained with hematoxylin and eosin. Arrows indicate hollow fibers. Scale bars, 500 µm. (**B**) The population of circulating white blood cells and serum concentrations of C-reactive protein (CRP). *P < 0.05 and **P < 0.01 vs. the PBS group. n = 8.

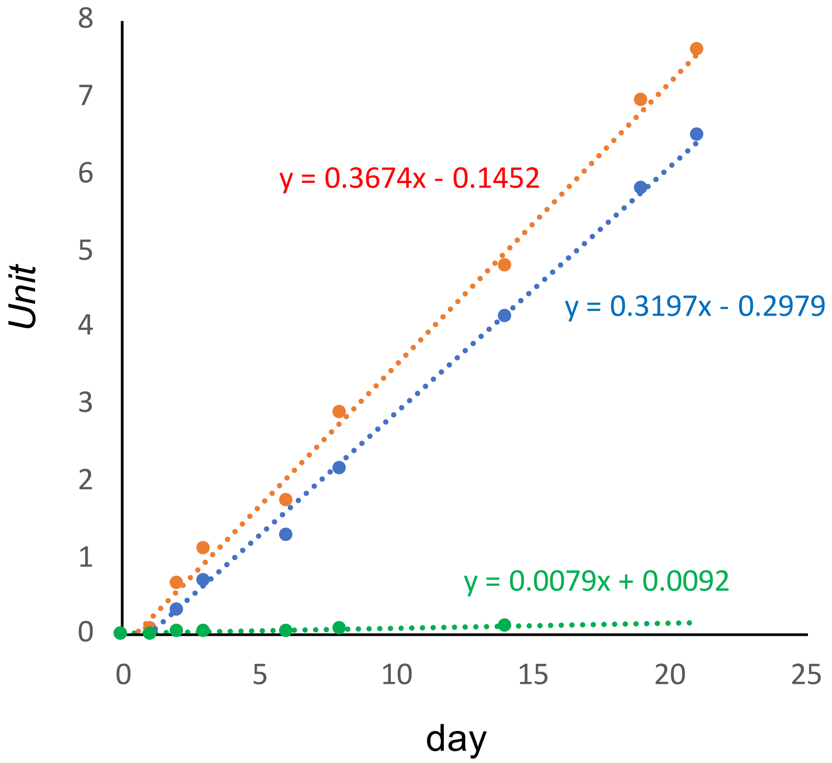

Supplementary Fig. 7 Cumulative insulin release kinetics from device under static state (no flow condition) *in vitro*. (Red) Release of Apidra (rapid-acting insulin) under 300 mg/dL glucose (n=1). (Blue) Release of Humulin (human recombinant insulin) under 300 mg/dL glucose (n=1). (Green) Release of Apidra under 100 mg/dL glucose (n=1). From slope of fitted lines, daily insulin dose can be estimated to be 367 mU/day, 320 mU/day and 8 mU/day, respectively, under these conditions.

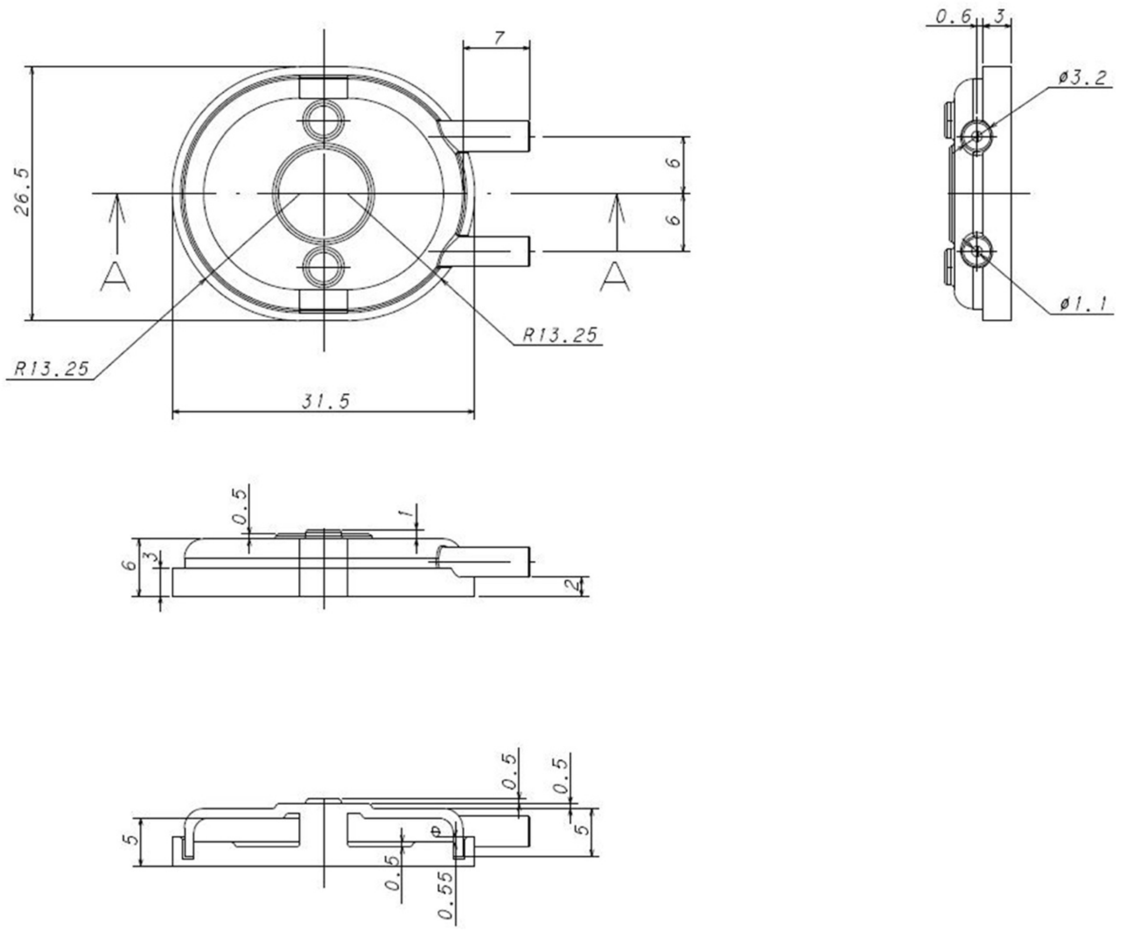

Supplementary Fig. 8. Design drawing of insulin reservoir.

Supplementary Table

Supplementary Tab. 1. Monomer molar fraction of hydrogels studied in SI Fig. 1. All figures have been rounded off to the second decimal place.

**Code**	**a**	**b**	**c**	**d**	**e**	**f**	**g**	**h**	**i**	**j**	**k**	**l**	**m**	**n**	**o**
NIPMAAm	0.93	0.75	0.63	0.54	0.56	0.48	0.41	0.35	0.42	0.36	0.31	0.23	0.36	0.23	0.14
FPBA	0.08	0.08	0.07	0.06	0.14	0.12	0.10	0.09	0.18	0.15	0.13	0.10	0.24	0.15	0.10
NHEAAm	0.00	0.17	0.30	0.41	0.30	0.41	0.49	0.56	0.41	0.49	0.56	0.68	0.41	0.62	0.76
